# Supplementary material for: Kernel Bioassay Evaluation of Maize Ear Rot and Genome-Wide Association Analysis for Identifying Genetic Loci Associated with Resistance to Fusarium graminearum Infection
Source: J Fungi (Basel). 2023 Dec 1;9(12):1157. doi: 10.3390/jof9121157 (PMC10744209; doi:10.3390/jof9121157)
Supplement: Supplementary file 1 [file jof-09-01157-s001.zip › jof-2699472-supplementary.pdf]

Supplementary Materials

Table S1. The 303 diverse maize inbred lines were evaluated for GER severity at 7, 14, 21 and 28 days post-inoculation (dpi) using kernel bioassay.

Material name	7 DPI (× 10^5)	14 DPI (× 10^5)	21 DPI (× 10^5)	28 DPI (× 10^5)	Mean (× 10^5)
**SCML1950**	**0.41**	**0.43**	**0.52**	**0.56**	**0.48**
**Qi533**	**0.53**	**0.81**	**0.58**	**0.17**	**0.52**
**JD7275**	**0.75**	**0.82**	**0.65**	**0.73**	**0.74**
**End28**	**0.46**	**1.52**	**0.45**	**0.79**	**0.81**
**5Gong**	**0.32**	**0.97**	**0.76**	**1.26**	**0.83**
**CG698C102**	**0.12**	**1.15**	**1.67**	**1.80**	**1.19**
**Su95-1**	**0.19**	**0.82**	**1.74**	**2.38**	**1.28**
**Lin-1**	**0.78**	**1.59**	**1.47**	**1.52**	**1.34**
**CLWN251**	**0.56**	**0.86**	**1.54**	**2.67**	**1.41**
**BJ005**	**0.47**	**0.33**	**2.32**	**2.69**	**1.45**
LZM009	1.22	0.95	1.41	2.46	1.51
CLYN223	0.44	2.41	1.68	1.53	1.52
Nan637	0.34	0.27	4.13	1.42	1.54
Lu2458	0.00	0.38	4.82	1.23	1.61
SW01D1058-5	0.54	4.10	2.18	0.00	1.71
CMY093288	0.47	1.48	2.15	2.72	1.71
XBY13563	0.45	3.23	0.36	3.39	1.86
XH05	0.27	0.59	6.91	0.48	2.06
CG921	2.81	0.37	0.95	4.32	2.11
M232	0.81	0.93	2.16	4.58	2.12
08WSC187	0.24	3.35	0.87	4.14	2.15
NZ013-1	0.00	8.89	0.00	0.00	2.22
CL02603	0.00	1.47	3.78	4.02	2.32
GP30-1	0.00	0.22	2.17	7.12	2.38
06WAM210	0.00	0.67	0.34	9.13	2.54
CLWN247	0.00	0.56	4.15	5.55	2.57
CL02720	0.00	0.00	9.67	0.65	2.58
Zheng28	0.26	1.83	0.37	8.11	2.64
CML308	0.24	3.51	6.31	0.58	2.66
Qi205	0.28	0.37	0.96	9.97	2.90
Dan3130	0.47	3.98	0.62	6.53	2.90
LX9801	0.32	0.47	0.23	10.60	2.91
08WSC51	0.65	0.33	8.76	2.14	2.97
CLWN250	0.44	1.55	7.85	2.56	3.10
ZP2012	1.48	1.26	0.36	9.85	3.24
HL5049	8.13	5.12	0.00	0.00	3.31
08WSC204	1.44	10.94	1.46	0.00	3.46
Y1027	0.92	3.35	8.58	1.56	3.60
Y1021	9.09	1.54	2.16	2.14	3.73
YA3237	4.46	2.56	0.26	8.22	3.88
141	3.45	1.24	3.35	7.50	3.89
7141_5	0.95	1.81	13.58	0.66	4.25
K169R	0.65	14.30	0.43	2.44	4.46

10WRB115	13.20	0.94	2.74	1.55	4.61
M14	7.84	2.45	4.52	3.75	4.64
JY01-3	0.25	15.30	1.25	2.07	4.72
Y8G	0.00	8.24	7.15	3.78	4.79
H10	0.00	17.56	2.47	0.25	5.07
L2010-3	1.41	4.81	2.15	12.46	5.21
646	1.58	16.59	1.84	0.87	5.22
Mian7317	1.90	3.34	12.27	4.55	5.52
BS08-565	0.00	0.95	15.60	5.75	5.58
Liao2379	1.45	5.15	4.25	11.78	5.66
2142	0.00	0.16	6.13	16.65	5.74
Mian723	7.45	10.35	2.75	2.55	5.78
PN0504-8	0.26	5.47	5.75	12.23	5.93
98009	0.00	0.32	4.78	19.12	6.06
Mian04185-4	1.78	5.17	7.42	10.55	6.23
PI43W	0.47	1.26	11.61	12.55	6.47
K102	2.16	8.32	3.35	12.71	6.64
434	0.48	0.38	6.34	19.54	6.69
1212638	0.00	0.00	13.53	14.17	6.93
Dong156	0.00	3.67	12.54	12.72	7.23
CG921	0.65	12.32	13.51	2.76	7.31
K22	1.19	2.25	1.87	24.59	7.48
Zheng99	6.47	9.17	6.79	7.56	7.50
M3-0	3.23	12.59	10.57	3.61	7.50
JH96B	1.86	6.35	10.15	12.28	7.66
Mian04185-8	3.37	1.86	21.93	4.57	7.93
KS001	1.85	22.23	6.93	1.39	8.10
TD1	1.45	24.80	3.96	3.55	8.44
7854	0.36	7.72	3.58	22.15	8.45
SCML2031	0.25	1.53	2.11	30.05	8.49
TL96B	0.00	1.56	0.39	32.25	8.55
M11	0.00	0.35	27.67	7.81	8.96
Y0827	4.69	3.69	1.25	26.92	9.14
Y1035	2.14	10.17	7.81	16.54	9.17
YS0	2.14	4.56	13.58	16.59	9.22
Dan340	6.23	4.27	9.98	16.52	9.25
81565	1.26	10.81	3.38	21.78	9.31
18-9-101	0.86	5.41	10.18	21.45	9.48
M165	10.43	3.96	5.75	18.72	9.72
GCML152	1.66	2.47	6.38	28.98	9.87
65232B	0.23	8.71	18.65	12.21	9.95
975-12	0.26	0.29	1.80	37.54	9.97
CLWN227	0.23	37.51	0.39	2.47	10.15
Su1611	2.81	3.64	7.52	27.87	10.46
9614	5.66	4.12	24.68	7.59	10.51
Nan202	4.15	10.55	25.57	1.87	10.54
Y1015	1.53	4.59	28.51	7.61	10.56
W7475	0.41	0.36	40.50	1.23	10.63
793	3.17	12.14	12.45	16.25	11.00

CLWN205	11.19	1.02	9.03	23.04	11.07
Nan09530	13.60	1.35	13.25	16.14	11.09
ML1108	6.18	5.17	13.28	20.05	11.17
10GY6057	3.36	0.96	7.58	33.16	11.27
Qi319	7.24	1.58	6.15	30.23	11.30
GD003	0.47	25.23	10.05	10.08	11.46
08WSC257	2.26	1.86	21.08	21.12	11.58
BML1228	18.12	7.85	18.80	2.01	11.70
DH3732	1.24	31.09	5.14	9.67	11.79
BML1269	6.43	4.16	2.01	35.01	11.90
Dan598	3.62	8.84	22.54	12.78	11.95
Du321	1.04	10.27	1.35	37.05	12.43
SW01D1058-2	0.80	9.30	0.30	40.00	12.60
510317	0.47	1.88	0.65	47.58	12.65
KS003	0.26	1.64	14.19	34.53	12.66
CML291	0.43	7.22	2.77	40.46	12.72
CLWN226	6.04	0.62	2.01	42.51	12.80
K305	7.10	16.55	15.13	12.68	12.87
S273	6.12	18.34	4.56	22.57	12.90
21209	3.23	27.55	2.73	18.26	12.94
LS-22	2.83	22.54	5.16	22.57	13.28
Y1127	0.84	9.09	3.96	40.12	13.50
LH7556	3.84	7.57	10.22	32.53	13.54
9LB050	2.84	28.51	13.22	10.58	13.79
QA	0.23	37.56	15.37	3.12	14.07
L31	0.00	34.15	15.96	7.89	14.50
TL98A1709-20	0.27	21.03	0.67	37.05	14.76
Zheng22	27.19	2.11	28.54	1.32	14.79
ZY2247	0.49	4.03	50.12	4.57	14.80
Zheng29	12.27	15.09	10.05	22.59	15.00
Yu561	1.56	13.57	42.14	3.06	15.08
LZM025	1.46	2.83	21.57	34.58	15.11
CLYN214	4.02	22.38	16.05	18.27	15.18
Shen137	0.00	0.00	0.48	60.36	15.21
LC955	1.53	24.06	34.57	1.11	15.32
Yu9537	0.44	24.37	4.28	32.18	15.32
09YT20919	4.46	15.26	28.51	13.53	15.44
SCML203	6.08	13.02	27.35	16.05	15.63
Nan21-3	3.17	40.56	10.26	9.03	15.76
18-599	0.41	1.24	31.56	31.57	16.20
Shen136	0.25	2.75	45.91	16.15	16.27
178	0.65	14.74	42.64	7.62	16.41
C2010-3	2.61	46.51	17.76	0.37	16.81
BANTAN2003	0.25	27.36	27.57	13.59	17.19
PHW65	1.82	3.22	31.56	32.57	17.29
De12	10.15	31.58	22.51	6.06	17.58
SCML2054	3.84	30.74	10.05	27.09	17.93
10GY76-111	2.87	1.28	10.52	57.46	18.03
Y0826	18.16	36.23	7.05	12.06	18.38

ZYDH381-1	18.37	20.37	30.23	5.13	18.53
B73	1.25	16.12	18.07	39.09	18.63
W8034	28.16	12.37	21.03	13.01	18.64
LSC107	0.24	13.15	28.15	33.41	18.74
Y1224	6.02	33.32	34.05	2.52	18.98
GCML57	16.03	34.05	14.07	12.23	19.10
Ji477	2.25	18.16	48.03	8.01	19.11
LSC127	0.27	0.86	30.01	45.34	19.12
BML1243	4.88	12.07	57.17	2.55	19.17
C24	3.76	7.38	10.55	55.58	19.32
CA211	1.55	3.16	6.39	67.72	19.71
CML379	4.04	9.09	27.12	40.05	20.08
PH4CV	1.02	6.25	21.18	52.04	20.12
5311	0.83	3.33	52.56	24.05	20.19
BML1275	0.00	0.37	75.62	5.72	20.43
LH8012	0.00	0.00	3.33	80.21	20.89
XBY2193	0.45	2.72	10.81	70.07	21.01
77	0.25	15.89	6.67	61.58	21.10
Ji1037	0.27	3.34	14.17	70.01	21.95
08WSC237	2.16	1.88	51.20	33.17	22.10
DH29	40.11	1.05	11.11	36.23	22.13
LX350	3.14	45.23	37.52	6.35	23.06
78599-211	50.24	6.18	33.09	3.06	23.14
LM-6	1.84	60.28	25.57	5.44	23.28
H127RE	1.44	0.66	27.17	68.27	24.39
LX312	1.96	3.65	3.03	90.58	24.81
21A	0.22	18.37	11.01	69.85	24.86
C09-1	28.34	1.28	21.28	49.57	25.12
YA8201	7.25	28.16	60.18	5.05	25.16
W8199	3.12	10.15	15.02	74.37	25.67
698-3	4.63	67.57	1.59	30.92	26.18
GP66-1	12.59	25.55	0.93	65.73	26.20
Liao6082	0.43	0.62	51.95	52.53	26.38
CTL26	0.64	11.76	78.26	16.56	26.81
ShuangM9	1.27	27.02	30.74	49.15	27.05
ZJ-3	0.44	36.63	58.05	15.84	27.74
1217 8107	2.53	42.93	27.36	40.05	28.22
Mian715	10.05	20.16	36.37	48.36	28.74
4011	51.83	57.73	2.15	4.28	29.00
K363	3.66	4.58	19.53	88.84	29.15
SAM3001	22.05	25.55	40.52	28.57	29.17
1323	5.28	3.92	46.55	61.52	29.32
Dan599	1.18	13.05	93.94	9.81	29.50
811	4.84	2.26	15.58	96.06	29.69
LJS-1	5.41	21.19	43.51	49.54	29.91
SCML202	57.28	40.55	2.04	20.36	30.06
PHV63	15.73	6.37	100.29	0.44	30.71
TY30331-3	36.16	33.38	36.47	17.56	30.89
ZD808-1	1.65	28.58	6.12	88.05	31.10

Y0921	0.23	1.28	5.72	120.36	31.90
Liao68	1.47	1.84	7.57	117.53	32.10
P801	0.65	10.08	36.34	82.56	32.41
XS021	0.00	0.23	22.85	107.52	32.65
H08-155	1.27	21.95	43.05	64.51	32.70
SH15	1.23	90.59	2.48	39.13	33.36
W284	3.16	7.52	13.56	110.01	33.56
Lian87	0.82	16.48	108.05	11.14	34.12
17564	0.23	1.65	32.88	102.54	34.33
GCML140	2.38	13.29	86.63	35.63	34.48
LZM004	38.84	20.41	18.15	60.74	34.54
Liao7996	6.20	5.16	22.05	105.94	34.84
10GY92-121	1.64	4.82	12.21	120.73	34.85
SN8-1-1	12.37	15.16	110.37	4.05	35.49
S7913	1.22	30.67	13.02	100.45	36.34
LN8	0.69	31.42	112.82	4.63	37.39
7327	18.15	12.26	88.05	32.35	37.70
1572	4.21	24.35	51.54	76.53	39.16
Wa138	0.12	4.54	85.57	66.56	39.20
975-13	0.00	0.00	19.05	138.07	39.28
Liao7890	1.63	0.98	0.32	155.46	39.60
Ye478	2.64	10.85	76.32	70.27	40.02
10WRC64	0.68	5.72	13.25	140.54	40.05
Y1005	4.24	50.47	60.46	47.39	40.64
U8112	0.00	57.14	106.05	0.53	40.93
CML268	26.47	90.13	13.54	34.05	41.05
35855	13.37	49.05	39.74	62.05	41.05
L6201	4.94	7.83	142.05	11.73	41.64
HuangC	4.26	5.21	155.45	2.76	41.92
MH9	19.01	9.16	24.23	116.01	42.10
V37	9.01	90.12	8.17	61.15	42.11
P953	40.46	25.58	40.46	68.54	43.76
PB80	7.12	6.13	102.02	64.52	44.95
06WAM110	2.64	22.15	82.05	75.98	45.71
Mo17	3.18	1.18	8.01	171.15	45.88
SAM31152A	2.41	44.15	81.02	57.42	46.25
Nan381	22.05	5.06	105.11	54.22	46.61
CML451	1.08	58.50	20.34	107.15	46.77
SW01D1058-7	4.02	30.00	147.05	6.13	46.80
CN9802	9.08	63.17	23.44	94.35	47.51
08WSC179	0.18	0.23	0.33	192.50	48.31
HZ127-7	40.05	140.23	5.71	10.05	49.01
03FLUSA10	8.11	21.43	109.05	60.91	49.88
BML1234	1.12	11.01	34.05	155.13	50.33
871	3.02	27.34	140.57	32.51	50.86
F06	3.22	6.07	9.24	185.13	50.92
CLRCY034	6.64	5.14	18.02	175.11	51.23
ZH64	6.60	52.54	87.75	63.63	52.63
Ji992	0.00	6.15	9.09	200.47	53.93

BML1256	11.09	24.23	52.05	130.25	54.41
En1824	21.23	56.05	21.10	120.62	54.75
Y1038	1.58	1.22	45.94	170.55	54.82
CML447	25.12	150.26	8.18	38.03	55.40
SW01D1031-14	17.13	40.05	124.16	45.35	56.67
SCML103	5.06	5.10	27.11	190.36	56.91
81565	0.65	5.47	23.21	198.85	57.05
Q78	10.64	0.44	166.58	55.26	58.23
W8071	15.03	105.05	78.23	38.04	59.09
LZM05-1-1	6.02	4.02	135.36	94.57	59.99
Y1216	0.67	8.47	3.09	234.58	61.70
618	11.47	6.19	51.46	182.05	62.79
JS0251	13.36	24.26	6.13	220.36	66.03
Shen135	1.18	2.37	203.40	60.53	66.87
98WV9	60.75	30.47	15.47	164.52	67.80
LLF-08	0.27	6.68	167.52	102.53	69.25
B151	0.44	4.58	126.54	147.05	69.65
Liao147-8	6.23	73.05	37.53	162.51	69.83
QBII-1	6.85	50.62	147.12	77.51	70.53
ZD0502-23111	4.07	137.52	13.54	130.75	71.47
CD30M	7.06	55.15	84.53	142.53	72.32
Y1217	1.95	31.15	205.81	60.23	74.79
9782	21.92	7.05	190.15	80.15	74.82
PHW52	72.74	27.93	60.57	147.94	77.30
2369	2.02	60.36	27.37	228.74	79.62
M89	12.03	65.64	0.61	241.15	79.86
P138	1.08	37.52	182.58	115.74	84.23
Y1018	14.36	30.46	217.59	79.52	85.48
F19	6.93	148.25	96.82	90.01	85.50
TLL-1	48.27	126.53	8.07	161.05	85.98
Yi99-19	0.41	12.27	324.95	15.15	88.20
08-641	5.12	34.15	272.17	48.73	90.04
9HT1804	0.00	18.09	0.62	364.95	95.92
ML1120	79.52	175.83	10.35	130.62	99.08
Zheng58	0.00	14.04	43.05	356.42	103.38
Y1111	9.14	30.15	344.38	42.85	106.63
HL5054	0.81	61.15	350.26	16.83	107.26
Liao3053	1.15	14.07	152.05	262.55	107.46
Y1032R	3.08	39.37	19.58	375.53	109.39
Jiao51	9.47	64.51	18.11	346.54	109.66
87916W	79.05	112.59	34.26	216.93	110.71
Y1022	37.52	18.16	94.36	345.95	124.00
07G83	1.11	192.05	196.74	118.26	127.04
TY30331-2	6.25	146.83	38.14	320.63	127.96
Dan4245	15.17	94.85	112.25	300.74	130.75
Y1032W	13.05	154.53	57.14	315.84	135.14
66781	1.02	49.50	267.53	250.18	142.06
CML282	49.14	43.26	180.64	316.25	147.32
W30	144.03	51.27	237.64	200.21	158.29

WZ-1	0.21	154.42	231.74	276.23	165.65
CA1108	2.25	248.53	155.63	265.25	167.92
CIMMYT-1	5.03	31.15	42.38	604.51	170.77
TS6278	0.00	18.64	60.95	610.23	172.46
JH59	0.28	152.15	420.37	148.74	180.39
4866	34.85	632.74	27.07	57.36	188.01
CLWN201	0.14	69.26	12.52	682.57	191.12
Jing07-4	32.64	51.95	14.43	665.74	191.19
08WSC200	5.80	640.04	124.14	19.56	197.39
MX714	6.36	117.25	224.08	465.35	203.26
JF52-3	5.02	45.67	390.01	374.20	203.73
GCML157	0.12	114.96	204.16	520.53	209.94
Chuan273	0.91	10.81	9.05	847.02	216.95
4379	1.02	160.01	486.37	250.14	224.39
08WSC166	23.09	82.56	1089.56	180.23	343.86

Supplementary Figure S1

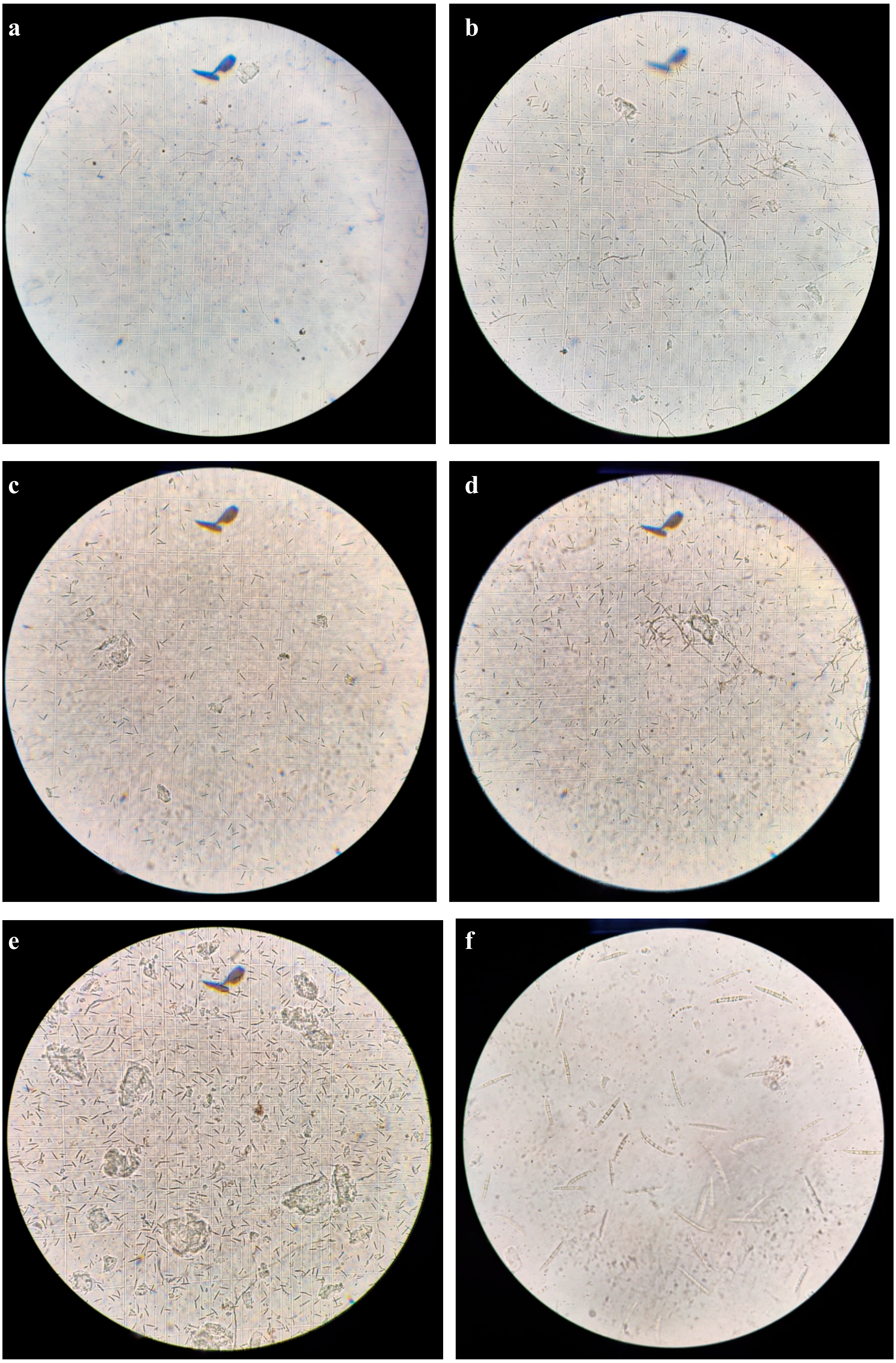

Supplementary Figure S1. Macroconidia characters of *Fusarium graminearum* at different time points. (**a**) 0 day post-inoculation (dpi). (**b**) 7 dpi. (**c**) 14 dpi. (**d**) 21 dpi. (**e**) 28 dpi. (**f**) Larger version of *Fusarium graminearum* macroconidia.
